# Process evaluation in drugs trials within adult learning disability populations: a systematic review

**DOI:** 10.1186/1745-6215-16-S2-P167

**Published:** 2015-11-16

**Authors:** Elizabeth Randell, Rachel McNamara

**Affiliations:** 1 Cardiff University, Cardiff, UK

## 

The majority of RCTs focus on outcomes rather than on the processes involved in implementing an intervention. Process evaluations within trials examine not only the implementation but also the receipt and setting of an intervention which aids the interpretation of outcome results. Currently, process evaluations tend to be the domain of ‘complex interventions’ in health and public health research rather than drugs trials (CTIMPs). CTIMPs tend to be thought of as more ‘simple’ interventions having linear pathways from the intervention to the outcome. However, as pharmacological interventions move past early stage research and are rolled out into practice, their implementation shifts to becoming complex in nature. This is particularly true when implementing interventions in populations where neurodevelopmental impairments mean an individual may lack capacity. Parents/carers looking after the best interests of such individuals effectively become extra ‘layers’ through which the intervention needs to be administered. This added degree of complexity is something which is perhaps not routinely addressed.

This systematic review is of drug trials that have been carried out in adult populations with neurodevelopmental impairments which incorporat a process evaluation element. A systematic search of PsycINFO, EMBASE, MEDline, CINAHL and the Cochrane Central Register of Controlled Trials will be undertaken. The aim being to identify existing methods of evaluating interventions and provide a qualitative narrative of these. Results will be used to develop the approach taken to later stage CTIMPs particularly in this population and to explore the potential for a new framework for evaluating interventions of this nature.

